# Glycemia Lowering Effect of an Aqueous Extract of *Hedychium coronarium* Leaves in Diabetic Rodent Models

**DOI:** 10.3390/nu11030629

**Published:** 2019-03-14

**Authors:** Ling-Shan Tse, Po-Lin Liao, Chi-Hao Tsai, Ching-Hao Li, Jiunn-Wang Liao, Jaw-Jou Kang, Yu-Wen Cheng

**Affiliations:** 1 School of Pharmacy, College of Pharmacy, Taipei Medical University, Taipei 110, Taiwan; zoetsels@gmail.com (L.-S.T.); d01447001@ntu.edu.tw (C.-H.T.); 2 Institute of Food Safety and Health Risk Assessment, School of Pharmaceutical Sciences, National Yang-Ming University, Taipei 112, Taiwan; plliao0825@gmail.com (P.-L.L.); jjkang@ym.edu.tw (J.-J.K.); 3 Institute of Toxicology, College of Medicine, National Taiwan University, Taipei 100, Taiwan; 4 Department of Physiology, School of Medicine, Graduate Institute of Medical Sciences, College of Medicine, Taipei Medical University, Taipei 110, Taiwan; bros22@tmu.edu.tw; 5 Graduate Institute of Veterinary Pathobiology, National Chung Hsing University, Taichung 402, Taiwan; jwliao@dragon.nchu.edu.tw

**Keywords:** *Hedychium coronarium*, type 2 diabetes, aldosterone, streptozotocin, metabolic syndrome, folk medicine

## Abstract

*Hedychium coronarium* has a long history of use worldwide as a food and in folk medicine. In this study, we aimed to investigate the effect of an aqueous extract of *H. coronarium* leaves (HC) on type 2 diabetes mellitus (T2DM). Two types of animal models were used in this study: Streptozotocin (STZ)-induced T2DM (Wistar rats; *N* = 8) and C57BKSdb/db mice (*N* = 5). After treatment with HC for 28 days, glucose tolerance improved in both of the diabetic animal models. As significant effects were shown after 14 days of treatment in the STZ-induced T2DM model, we carried out the experiments with it. After 28 days of treatment with HC, the levels of cholesterol, triglyceride, high-density lipoprotein, and low-density lipoprotein were significantly improved in the STZ-induced T2DM model. The lesions degree of islet β-cells was decreased after the HC treatment. Although the insulin level increased moderately, the aldosterone level was significantly decreased in the HC-treated groups, suggesting that aldosterone might play an important role in this effect. In summary, HC is a natural product and it is worth exploring its effect on T2DM.

## 1. Introduction

Diabetes is a chronic progressive disease and one of the ten leading causes of death worldwide [1]. In the past three decades, the prevalence of type 2 diabetes mellitus (T2DM) has increased dramatically in countries of all income levels. Over time, diabetes causes various complications: It starts by damaging blood vessels, reducing the blood flow, with sequelae that may be macrovascular (heart attack, stroke, and heart failure) [2,3,4], or microvascular (blindness [5,6,7] and kidney failure [8]), or causing neuropathies (lower limb amputation). When diabetes is not controlled, not only is the patient quality of life affected and present a burden on medical resources, but the condition may also lead to death. In 2015, the World Health Organization (WHO) declared diabetes as one of the four priority noncommunicable diseases (NCDs) and established a diabetes program to reduce the impact of diabetes by 2020. The management of diabetes consists of two main steps: Preventing (decreasing the possible risk factors) and stabilizing the disease progress (early diagnosis, medication, and intake management) [9]. To stabilize the disease, it is crucial to adopt effective measures for surveillance and treatment strategies (pharmacologic and non-pharmacologic interventions) [10,11]. In addition to the development of novel drugs, the use of traditional medicine and food supplements for the treatment of T2DM should be investigated.

Traditional medicine (also known as folk medicine) has been used globally for centuries. Although the medicinal properties of resources such as mollusks and plants rely on the inheritance of experience, they have been gradually accepted in modern medicine. A food supplement is a dosed formulation of food and herbs, and provides medical benefits through its biologically active components [12]. Therefore, both traditional medicine and food supplements can be part of the treatment strategies for diabetes.

Extensive research has focused on the rhizome of *Hedychium coronarium*. However, the pharmacological benefits of *H. coronarium* leaf appear overlooked. *H. coronarium* is highly accessible and its leaf is a common vegetable in Taiwan. Therefore, in this study, two types of animal models were used to evaluate the lowering blood glucose level benefits of an aqueous extract of *H. coronarium* leaves (HC). A new supplement was developed, called SugarOut (SO), which contained 15% red yeast rice (RYR) and 7.2% *H. coronarium*. Red yeast rice (RYR) is a fermentation product that is traditionally used in East Asia to dye and preserve food. Its main pharmacologically active compound is monacolin-K (also called lovastatin).

*H. coronarium* (also called ginger lily), a plant approximately 1–3 m in height and has a long history of use in food and traditional folk medicine. For example, it is used in beauty products in Hawaii and Japan, as an essential oil in Vietnam, and as a vegetable in Malaysia. It can help ease indigestion, inflammation, insomnia, and pain in the muscles, joints, and abdomen [13,14]. In Brazil, *H. coronarium* leaf is considered a diuretic [15] and is used for the treatment of hypertension [16]. In India, the rhizome is used for the treatment of diabetes [17]. Many different bioactive compounds have been isolated from *H. coronarium* and their pharmacological effects have been established. For example, diterpenoids and a diarylheptanoid showed anti-angiogenic activity and suppressed the growth of different cancer cell types [18]. Coronarin D shows active resistance to Gram-positive bacteria and fungi [19], induces G2/M arrest, apoptosis, and autophagy [20]. Hedychilactones A, B, and C inhibit increases in nitric oxide (NO) production and the induction of inducible NO synthase [21]. Quercetin-3-*O*-glucuronide (Q3GA) has been reported to show beneficial effects in the reduction and prevention of various diseases, including neurodegenerative diseases, and to exert anti-inflammatory and antioxidant activities [22].

## 2. Material and Methods

### 2.1. Preparation of Aqueous Extract of Hedychium coronarium and SugarOut

The HC (containing 1.4% Q3GA) was a deep brown powder. Fresh overground parts (leaves and stems) of *H. coronarium* were collected in Pingtung, Taiwan. The dried leaves and stems of *H. coronarium* (100 kg) were extracted in 100% water at room temperature (25–35 °C). The 100% water extracts were concentrated in vacuo and then lyophilized to obtain a dark brown powder (8.4% yield).

SO was a prototype supplement (dark red powder) developed from *H. coronarium* that assisted with blood glucose regulation. It contained two main extracts: The RYR extract (15%) and the HC (7.2%). The two main bioactive compounds were monacolin K 1.8 mg (±20%) and Q3GA 0.6 mg (±20%).

Both test substances were provided by Vinovo Inc. The HC was stored at 4 °C and the SO was stored at room temperature. Both test substances were freshly dissolved in distilled water and administered by oral gavage daily to all rodents in the morning.

### 2.2. Animal Study

The Streptozotocin (STZ)-induced T2DM model was established in Wistar rats obtained from BioLASCO Ltd. (Taipei, Taiwan) and C57BKS^db/db^ mice (termed db/db here) were obtained from the National Laboratory Animal Center (Taipei, Taiwan). All procedures involving the use of animals were in compliance with the Guide for the Care and Use of Laboratory Animals (Press, 1996) and approved by the Institutional Animal Care and Use Committee at our institution (Approval no. LAC-2016-0168). The preliminary data suggested that the effective dose of SO was 246 mg/kg in the db/db model. As bioavailability differs between rodent species, based on FDA Guidance, the conversion factors for the rat Wistar STZ model and the mouse db/db model were 6.2 and 12.3, respectively [23]. Therefore, 124 mg/kg SO was administered to the STZ-T2DM rats and 246 mg/kg was administered to the C57BKS^db/db^ mice by oral gavage.

#### 2.2.1. C57BKS^db/db^ Mice (db/db Model)

**Figure 1:**
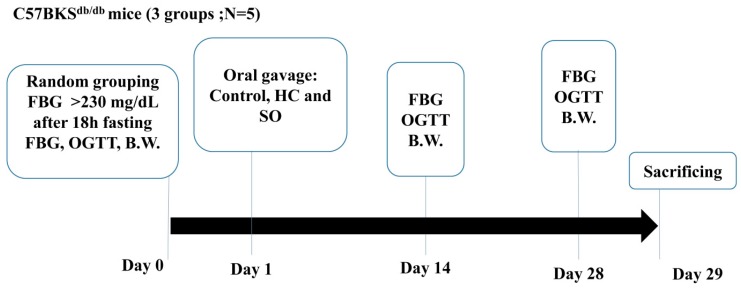
Study procedure for the C57BKS^db/db^ mice (termed db/db here). Fifteen male db/db mice were randomly divided into three groups (*N* = 5): Control, treatment with *Hedychium coronarium* leaves (HC), or SugarOut (SO) supplement. FBG, fasting blood glucose; OGTT, oral glucose tolerance test; B.W., Body Weight.

The C57BKS^db/db^ mouse is a model of diabetes with a spontaneous mutation (Lepr^db^) resulting in morbid obesity, chronic hyperglycemia, pancreatic beta cell atrophy, and low insulin [24]. The diabetes model is determined to be well-established when the blood glucose level is >230 mg/dL after 18 h fasting. Fifteen male mice (25–30 g) were divided into the following three groups (*N* = 5): Control (treated with distilled water); HC (17.71 mg/kg); and SO (246 mg/kg) [23] (Figure 1).

#### 2.2.2. STZ-Induced Type 2 Diabetes Model

**Figure 2:**
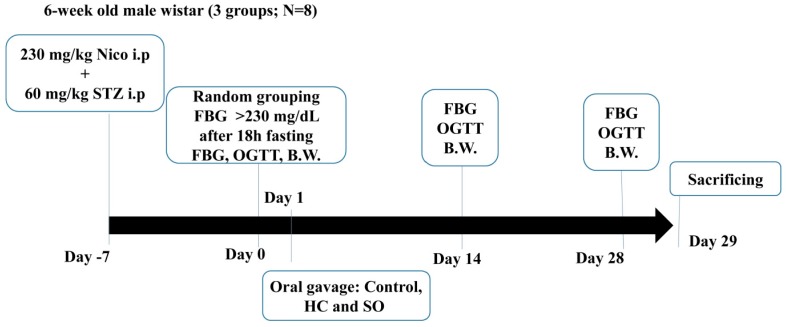
Study procedure for Streptozotocin (STZ)-induced type 2 diabetes rats. Diabetes was induced in 24 male Wistar rats, which were then randomly divided into three groups (Control, HC, and SO; *N* = 8).

T2DM was induced in 24 6-week-old male Wistar rats by the administration of 65 mg/kg STZ in 0.1 M citrate solution 15 min after nicotinamide injection (230 mg/kg in saline; intraperitoneally) [25,26,27,28]. The rats were caged (two animals per cage) in a controlled environment (12 h light/dark cycle, 23 ± 1 °C, and 39–43% relative humidity). The fasting glucose level was measured 1 week after the injections. The T2DM model was determined to be well-established when the blood glucose level was >230 mg/dL after 18 h of fasting. After T2DM was established, the rats were randomly divided into three groups of eight rats: Control; HC (8.928 mg/kg); and SO (124 mg/kg). An additional eight Wistar rats were used as the sham control (Figure 2).

### 2.3. Fasting Blood Glucose and Oral Glucose Tolerance Test

All rodents were administered the test substances daily. The body weight and fasting blood glucose (FBG) was measured on day 0, 14, and 28. The oral glucose tolerance test (OGTT) was performed on day 14 and 28. Each rodent fasted for 16 h before the FBG measurement. The test substances were administered 30 min before glucose challenge (1 g/kg), and then the blood glucose was tested 0, 30, 60, 90, and 120 min after the challenge by using IME-DC glucose test strips (IME-DC, Berlin, Germany).

### 2.4. Aldosterone and Insulin Levels

Serum samples were collected on day 29 after the T2DM rats were sacrificed. Insulin was quantified through the measurement of the optical density at 450 nm by using a Mercodia Ultrasensitive rat insulin ELISA kit (Mercodia AB, Uppsala, Sweden) and aldosterone was quantified through the measurement of the optical density at 405 nm corrected by the measurement at 590 nm by using an aldosterone ELISA kit (ab136933; Abcam, Cambridge, UK).

### 2.5. Histopathological Analysis

The pancreas was excised from T2DM rats after they were sacrificed on day 29. The samples were fixed in 10% neutral buffered formalin, and then prepared and examined by a professional pathologist from the Graduate Institute of Veterinary Pathobiology, National Chung Hsing University, Taichung, Taiwan. The severity of lesions in the islet β-cells of the pancreas was graded according to the methods described by Shackelford et al. [29]. The degree of lesions was graded from one to five, depending on severity: 1 = minimal (<1%); 2 = slight (1–25%); 3 = moderate (26–50%); 4 = moderately severe (51–75%); 5 = severe/high (76–100%).

### 2.6. Serum Biochemical Analysis

Blood samples from the T2DM rats were collected at the end of the 28-day oral administration period and then analyzed by using an Express Plus automatic clinical chemistry analyzer (Siemens Healthineers, Erlangen, Germany).

### 2.7. Statistical Analysis

All values are expressed as the mean ± standard deviation (SD) in tables and the mean ± standard error (SE) in figures. The comparisons between groups were performed by one-way analysis of variance (ANOVA) followed by Scheffe multiple comparison tests using SPSS Statistical Software (IBM, New York, NY, USA). Values of *p* < 0.05 were considered significant.

## 3. Results

### 3.1. HC Improved Fasting Blood Glucose and Glucose Tolerance in Both Diabetic Animal Models After 28 Days of Treatment

**Figure 3:**
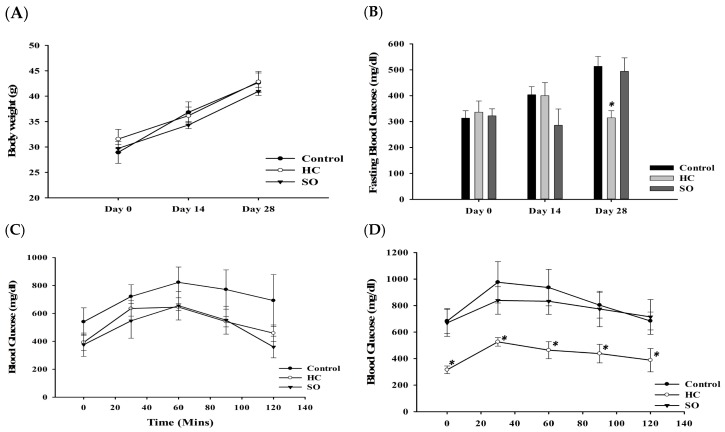
Effects of HC and SO in C57BKS^db/db^ mice (*N* = 5). (**A**) Changes in body weight. (**B**) Changes in fasting blood glucose (FBG) after treatment with HC and SO for 14 days and 28 days. (**C**) Oral glucose tolerance test (OGTT) after administration of HC and SO for 14 days. (**D**) OGTT after administration of HC and SO for 28 days. Significant difference between the control-treated group at * *p* < 0.05, by one-way ANOVA.

In the db/db model, after oral gavage of SO and HC, mice gained weight slower than the control group (Figure 3A). The HC decreased the FBG (Figure 3B) and increased the glucose tolerance after treatment for 14 days (Figure 3C); a significant difference was observed after treatment for 28 days (Figure 3D). SO also affected the FBG and glucose tolerance, however, the effects of SO were not as remarkable as those of the HC.

**Figure 4:**
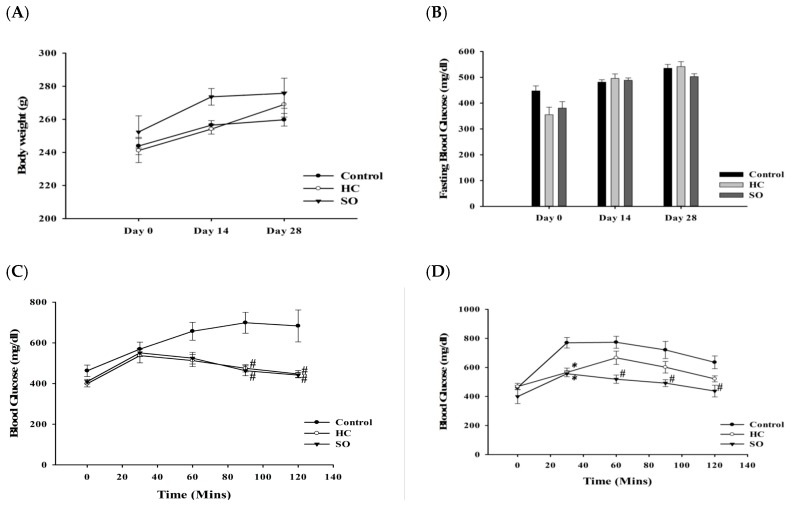
STZ-induced type 2 diabetes model (T2DM; *N* = 8) after the administration of HC and SO for 14 and 28 days. (**A**) Changes in body weight. (**B**) Changes in fasting blood glucose. (**C**) OGTT after administration of HC and SO for 14 days. (**D**) OGTT after administration of HC and SO for 28 days. Significant difference between control-treated group at * *p* < 0.05, # *p* < 0.01 by one-way ANOVA.

In general, the STZ-induced T2DM model shows a significant weight reduction [30]. In this study, after oral gavage of SO and HC for 28 days, the body weights of mice receiving HC and SO was increased compared to that in the control group (Figure 4A) and the FBG was slightly lower in the SO group on day 28 (Figure 4B). After 14 days of oral administration of HC and SO, the blood glucose level in the HC and SO groups was significantly decreased compared with that in the control group at 60, 90, and 120 min after intake of 1 g/kg glucose (Figure 4C; *p*-value in Supplementary Table S1). After administration of HC and SO for 28 days (Figure 4D), the glucose level in the SO group was significantly lower than that in control group from 30 to 120 min after the intake of 1 g/kg glucose (*p*-value in Supplementary Table S1), however, the HC only resulted in a significant difference at 30 min after glucose intake. The area under the curve (AUC) of both the SO and HC groups was significantly lower than that of the control group (Table 1). These results suggested that HC could improve the FBG and glucose tolerance after 28 days in the db/db mice. A notable increase in glucose tolerance was observed in both the HC and SO groups after 14 days of administration, and a significant increase in glucose tolerance was observed after 28 days of administration in the T2DM model. Therefore, the following experiments focused on the STZ-induced T2DM model.

### 3.2. H. coronarium Attenuated STZ-Induced Pancreatic Damage and Ameliorated the Markers of Metabolic Syndrome

**Figure 5:**
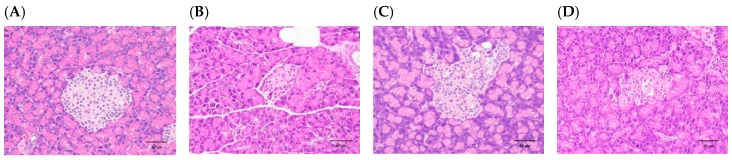
Histopathological changes by hemotoxylin and eosin (H & E) staining (400×) of the islets of the pancreas in rats with STZ-induced β-cell toxicity. (**A**) Sham control. Normal architecture of the β-cells in the islets of the pancreas. STZ induced a slight to moderate/severe decrease of β-cells in the islets of the pancreas in (**B**) T2DM control model. (**C**) T2DM model treated with HC for 28 days. (**D**) T2DM model treated with SO for 28 days. Scale bar = 50 μm.

**Figure 6:**
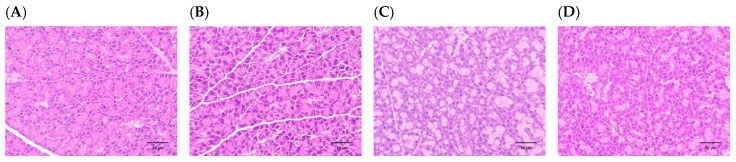
Histopathological changes (H & E staining, 400×) of the acinar cells in the pancreas in rats with STZ-induced β-cell toxicity. (**A**) Sham control. Normal architecture of the β-cells in the islets of the pancreas. STZ induced a slight to moderate/severe decrease of β-cells in the islets of the pancreas in (**B**) T2DM control model. (**C**) T2DM model treated with HC for 28 days. (**D**) T2DM model treated with SO for 28 days. Scale bar = 50 μm.

The levels of cholesterol, triglyceride, high-density lipoprotein (HDL), and low-density lipoprotein (LDL) were significantly decreased in the STZ-induced T2DM model compared with the control group. In the HC group, creatinine and blood urea nitrogen (BUN) were lower than the control group and similar to the sham group. The SO group did not show any significant differences compared with the control group, but the laboratory results tended to be similar to those in the sham group (Table 2). STZ caused a severe decrease in islet β-cells (Figure 5B) and severe atrophy of acinar cells in the pancreas (Figure 5B; Figure 6B). Biopsy sections of the HC (Figure 5C; Figure 6C) and SO (Figure 5D; Figure 6D) groups showed that the morphology of the islet β-cells and acinar cells of the pancreas tended to be similar to the sham group (Figure 5A; Figure 6A). According to the degree of lesions (Table 3) after 28 days treatment, HC (0.79-fold) and SO (0.68-fold) prevented the decrease of islet cells and the atrophy of acinar cells (HC, 0.85-fold; SO 0.70-fold). HC and SO had moderate protective effects against the damage caused by STZ and regulated lipid markers (Table 2 and Table S2). However, the mechanism underlying the beneficial effects of HC on T2DM remains to be elucidated.

### 3.3. HC Altered Insulin and Aldosterone Content in Blood

**Figure 7:**
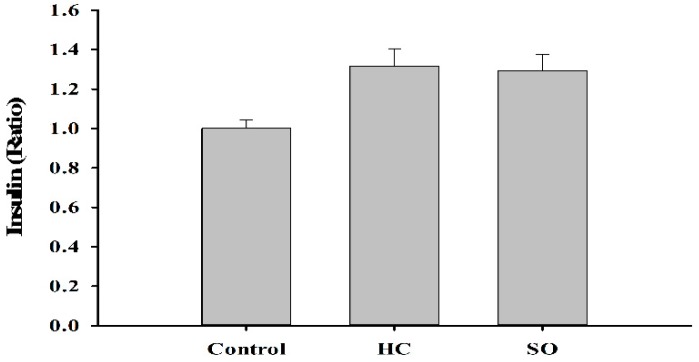
Insulin level in T2DM model rats after 28 days of treatment. No significant difference was found between the control-treated group by one-way ANOVA.

**Figure 8:**
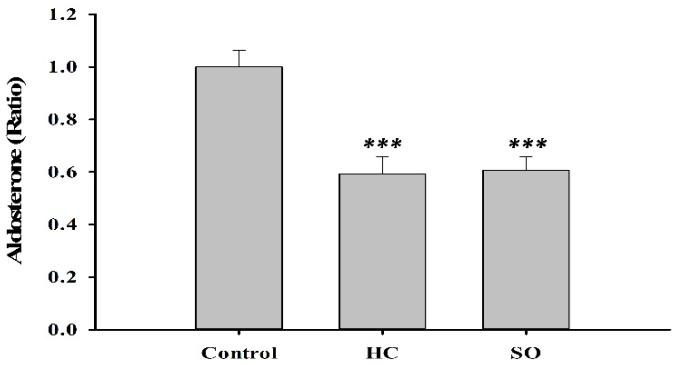
Aldosterone level in T2DM model rats after 28 days of treatment. Significant difference between the control-treated group at *** *p* < 0.001 by one-way ANOVA.

Insulin levels in the HC (1.32-fold) and SO (1.29-fold) groups increased moderately compared with the control group (Figure 7). Much research has shown that the renin-angiotensin-aldosterone system (RAAS) plays a critical role in diabetes [31,32,33]. In our previous study, the oral administration of *H. coronarium* aqueous extract (3 g/kg) to Sprague Dawley rats for 90 days, resulted in a decrease of aldosterone levels in the serum. Therefore, we measured the aldosterone level in the present study. Aldosterone was significantly decreased in both HC (0.59-fold) and SO (0.61-fold) groups compared with that in the control group (Figure 8).

## 4. Discussion and Conclusions

In this study, we aimed to explore a natural product, *H. coronarium*, and determine if it would benefit people with diabetes. Two types of diabetic rodent models were used to determine the glycemia lowering effect of an aqueous extract of *H. coronarium* leaves. We found that HC significantly increased glucose tolerance in both diabetic models, improved the lipid profile, moderately increased insulin, benefited β-cell structure, and decreased the aldosterone level in an STZ-induced T2DM model. Although HC has been used as a folk medicine worldwide—as a diuretic and for the treatment of inflammation, hypertension, and diabetes—its mechanism of action is yet to be elucidated.

Previous studies have reported that the RAAS has played a major role in diabetes; aldosterone is significantly increased in primary hyperaldosteronism, diabetes, and other metabolic syndromes. An increase in aldosterone causes impaired glucose tolerance, decreased pancreatic β-cell function, and tissue insulin sensitivity [31,34,35]. Aldosterone is a mineralocorticoid hormone that is produced from cholesterol in the cortex of the adrenal gland. It interacts with the mineralocorticoid receptor (MR) to regulate blood pressure, water sodium, and potassium homeostasis [36]. In addition, it has genomic and non-genomic actions. The genomic actions occur through the binding of aldosterone to cytoplasmic MR, and the aldosterone-MR complex translocates to the nucleus and modulates nuclear transcription [37]. As for the rapid non-genomic action, aldosterone increases intracellular Ca^2+^ and protein kinase C (PKC) activation. In addition, aldosterone activates and stimulates Na^+^/K^+^-ATPase, Na^+^/K^+^/2Cl^+^, NHE1, and NBCe1 [38,39,40], and other pathways, such as the Mitogen-activated protein (MAP) kinases pathway, adenylate cyclase, tyrosine kinase, and cAMP-dependent protein kinase. Therefore, understanding the complete regulation of aldosterone biosynthesis will allow medicinal interventions for the management of hypertension, congestive heart failure, renal disease, and diabetes mellitus [41]. In this study, both HC and SO significantly decreased aldosterone levels and increased glucose tolerance in the STZ-induced T2DM model. These findings suggest that T2DM may be improved by alteration of the aldosterone levels, but more studies are recommended to understand the regulation of this pathway.

Flavonoids may play a role in many metabolic processes involved in T2DM, and Q3GA (also known as miquelianin) is a flavonol glucuronide. Q3GA has been proven to inhibit the production of reactive oxygen species (ROS), low-density lipoprotein (LDL) oxidation [42,43], act as an anti-inflammatory, and improve insulin resistance in skeletal cells [44]. Q3GA also inhibited angiotensin II (Ang II)-induced increases in the DNA binding activity of activator protein (AP)-1, a downstream transcription factor of c-Jun N-terminal kinases (JNK), composed of the c-Jun homo/heterodimer [45]. Angiotensin II interacts with the angiotensin receptor (AT1) membrane receptor that is coupled to cellular second messengers, it is important in the regulation of aldosterone secretion [46]. As Q3GA can reduce the effect of Ang II, it may also contribute to the regulation of aldosterone.

As we would like to develop a new supplement containing HC, we also examined the effect of SO in this study. RYR has been reported to exert anti-inflammatory, hypotensive, cholesterol-lowering, cardioprotective, anticancer, and osteogenic activities [47]. In a previous study, RYR extract (300 mg/kg/day) was reported to decrease the FBG, increase insulin secretion, and protect islet cells in db/db mice [48]. Therefore, we selected RYR for the development of a food supplement containing HC. When the two compounds were combined, we expected an additional effect. In the present study, we also tested the efficacy of SO. SO slightly lowered fasting blood glucose after 28 days of treatment in the STZ -induced T2DM model (Figure 4B). The glucose tolerance (Figure 4C,D) in the SO treatment group was moderately increased compared with that in the HC group after 28 days. However, no differences in insulin, aldosterone, and histopathological findings were observed when the SO group was compared with the HC group. We found that HC exerts beneficial effects in diabetes through modulation of the aldosterone level in the blood to improve glucose tolerance. SO may slightly assist in the improvement of glucose tolerance, although the supplement formula requires improvement. SO also showed effects in two animal models, however, the efficacy of SO in the db/db model was not as strong as HC. The db/db model is known to have as defects in the leptin receptor, leading to increases in insulin and blood glucose, insulin resistance, and obesity [49]. In contrast, STZ is a DNA alkylating agent that targets β-cells [25,26,27,28]. As shown by the lipid profile in the STZ model, SO has the ability to ameliorate lipid markers, although not to the same extent as HC (Table 2). This explained how SO lowered the FBG and increased the glucose tolerance after 14 days of treatment, but was not as effective as HC during day 28 in db/db model (Figure 3).

In summary, we treated two types of diabetic rodent models with HC and SO, and found that both HC and SO exerted beneficial effects on T2DM. However, SO treatment did not show a significant difference compared with the HC treatment. The underlying mechanisms of HC, and the interactions of HC and RYR combined, are not wholly investigated yet. Therefore, we suggest that HC could be a suitable candidate for the development of drugs and food supplements for the treatment of T2DM, but more studies should be performed in order to understand the profound mechanism.

## Figures and Tables

**Table 1:** Area under the curve (AUC) of blood glucose in the oral glucose tolerance test (OGTT).

AUC	14 Days	28 Days
Sham	8898.75 ± 1816.12	1666.88 ± 3399.66
Control	23,578.13 ± 9636.76	32,130.00 ± 8133.19
*H. coronarium* leaves (HC)	11,730.00 ± 5959.70	14,979.38 ± 5656.03 *
SugarOut (SO)	10,301.25 ± 5884.57	11,945.63 ± 13,782.89 **

Sham = wild-type Wistar rats treated with distilled water. *N* = 8 in each group. The data are expressed as the mean ± standard deviation (SD). Significant difference between the control-treated group at * *p* < 0.05, ** *p* < 0.01 by one-way ANOVA.

**Table 2:** Biochemical analysis of T2DM rat model after treatment with HC and SO for 28 days.

Group		Sham	Control	HC	SO
Number of Animals		8	8	8	8
Cholesterol	mg/dL	69.28 ± 25.71	161.50 ± 63.79	76.63 ± 15.30 **	111.63 ± 51.48
Triglycerides	mg/dL	58.13 ± 23.93	753.38 ± 434.92	105.13 ± 33.31 **	480.75 ± 410.14
HDL	mmol/L	27.13 ± 12.16	33.38 ± 13.23	42.75 ± 13.10	38.50 ± 12.31
LDL	mmol/L	7.63 ± 1.77	37.63 ± 19.83	10.50 ± 2.56 **	22.25 ± 21.02

HDL: low-density lipoproteins cholesterol; LDL: low-density lipoproteins cholesterol. Data are expressed as the mean ± SD. Significant difference between the control-treated group at ** *p* < 0.01 by one-way ANOVA.

**Table 3:** Degree of lesions in the pancreas of the T2DM model animals after treatment with HC and SO for 28 days.

Pancreas	Control	HC	SO
Decrease, β-cell, islet, focal	3.80 ± 0.45	3.00 ± 0.71	2.60 ± 0.55
Atrophy, acinar cell, diffuse	4.00 ± 0.00	3.40 ± 0.89	2.80 ± 1.10

The degree of lesions was graded from one to five depending on severity: 1 = minimal (<1%); 2 = slight (1–25%); 3 = moderate (26–50%); 4 = moderate/severe (51–75%); 5 = severe/high (76–100%).

## Supplementary Materials

The following are available online at https://www.mdpi.com/2072-6643/11/3/629/s1, Table S1: *p*-Value of oral glucose tolerance test (OGTT) after administrating *Hedychium coronarium* (HC) and SugarOut (SO) in STZ-induced type 2 diabetes model (T2DM; *N* = 8); Table S2: Biochemistry analysis of STZ-T2DM after treating HC and SO for 28 days.

## Abbreviations

T2DM	type 2 diabetes;
HDL	high-density lipoproteins cholesterol;
LDL	low-density lipoproteins cholesterol;
OGTT	oral glucose tolerance test’
FBG	fasting blood glucose
