# Supplementary material for: Glycemia Lowering Effect of an Aqueous Extract of Hedychium coronarium Leaves in Diabetic Rodent Models
Source: Nutrients. 2019 Mar 14;11(3):629. doi: 10.3390/nu11030629 (PMC6470712; doi:10.3390/nu11030629)
Supplement: Supplementary file 1 [file nutrients-11-00629-s001.pdf]

nutrients

MDPI

Supplementary Materials:

Table S1. *p*-Value of oral glucose tolerance test (OGTT) after administrating *Hedychium coronarium* (HC) and SugarOut (SO) in STZ-induced type 2 diabetes model (T2DM; *N* = 8).

t (min)	0	30	60	90	120
14 days					
Control	-	-	--	-	-
HC	-	-	-	0.001	0.007
SO	-	-	-	0.001	0.005
28 days					
Control	-	-	-	-	-
HC	-	0.017	-	-	-
SO	-	0.011	0.003	0.007	0.042

Table S2. Biochemistry analysis of STZ-T2DM after treating HC and SO for 28 days.

Group		Sham	Control	HC	SO
Number of animals		8	8	8	8
Calcium	mg/dL	10.18 ± 0.36	9.03 ± 1.19	9.29 ± 0.62	8.66 ± 0.90
Chloride	mEg/L	97.63 ± 3.96	83.13 ± 6.49	85.75 ± 3.37	85.75 ± 3.65
Phosphorus	mg/dL	7.43 ± 4.48	11.31 ± 6.55	8.80 ± 1.11	8.31 ± 3.34
Potassium	mEg/L	9.34 ± 4.21	18.44 ± 14.86	20.21 ± 3.49	15.15 ± 7.74
Sodium	mEg/L	142.75 ± 6.36	123.38 ± 10.99	123.00 ± 3.02	127.00 ± 7.21
Magnesium	mg/dL	2.88 ± 0.58	2.75 ± 0.60	2.49 ± 0.36	2.65 ± 0.31
ALT	U/L	35.25 ± 10.44	92.38 ± 58.40	80.88 ± 44.88	77.88 ± 23.17
AST	U/L	127.25 ± 54.76	156.13 ± 117.04	197.50 ± 98.55	162.63 ± 58.39
ALP	U/L	73.88 ± 21.16	397.75 ± 60.35	309.25 ± 121.27	446.13 ± 120.87
γ-GT	U/L	3.00 ± 0.00	4.75 ± 2.87	4.13 ± 1.81	4.13 ± 1.64
T-Bil	mg/dL	0.20 ± 0.00	0.25 ± 0.14	0.20 ± 0.00	0.20 ± 0.00
Creatinine	mg/dL	0.44 ± 0.17	0.75 ± 0.39	0.41 ± 0.06	0.56 ± 0.27
BUN	mg/dL	17.88 ± 4.36	48.75 ± 13.26	29.13 ± 9.34	47.88 ± 20.73
Total protein	g/dL	5.96 ± 0.29	5.15 ± 0.85	5.11 ± 0.46	5.01 ± 0.23
Albumin	g/dL	4.10 ± 0.58	3.33 ± 0.34	3.63 ± 0.30	3.60 ± 0.29
Globulin	g/dL	1.86 ± 0.47	1.83 ± 0.96	1.49 ± 0.33	1.41 ± 0.28

ALT: alanine aminotransferase; AST: aspartate aminotransferase; ALP: Alkaline phosphatase; γ-GT: Gamma-glutamyl transferase; T-Bil: Total Bilirubin; BUN: blood urea nitrogen. Data were expressed as Mean ± S.D.
